# Adult-Onset Nephrotic Syndrome in the Older Adults: A Case Series

**DOI:** 10.7759/cureus.98181

**Published:** 2025-11-30

**Authors:** Nikhil Choudhary, Kritartha Kashyap, Pankhuri Saxena, Sudeep M George, Minakshi Dhar

**Affiliations:** 1 Geriatric Medicine, All India Institute of Medical Sciences, Rishikesh, Rishikesh, IND

**Keywords:** adult nephrotic syndrome, case-series, geriatric medicine, glomerulonephritis (gn), older adult, renal biopsy in nephrotic syndrome

## Abstract

In older adults, glomerular diseases are becoming increasingly recognized due to increased longevity and an ageing population. Diagnosis of nephrotic syndrome is often missed or underdiagnosed due to relative inertia for subjecting them to renal biopsies. The spectrum of nephrotic syndrome in this group is varied, with different therapeutic strategies and prognosis, often complicated by the interplay of multi-morbidity and age-related changes. In this case series, we report three older patients with nephrotic syndrome diagnosed with different aetiologies.

## Introduction

Nephrotic syndrome is one of the pathognomonic clinical presentations in the spectrum of glomerular nephropathy that classically presents with the pentad of proteinuria (>3.5 grams/day), hypoalbuminemia (<3.5 g/dL), oedema, lipiduria and hypercholesterolemia [1]. The prevalence of nephrotic syndrome in the geriatric population ranges from 30 to 62.5%, in patients who have undergone renal biopsies for various causes, including proteinuria, haematuria or unknown renal dysfunction [2,3]. Similarity of clinical presentation with other medical conditions, such as congestive heart failure, and geriatric syndromes like frailty and sarcopenia, age-related structural and functional decline in the renal system and clinical inertia for invasive tests like renal biopsy can delay its diagnosis.

As there is a limited understanding of glomerulonephritis in older adults due to scarcity of literature and only a small percentage being subjected to biopsies, we report here a series of three cases who presented with similar clinical scenarios but with different underlying aetiologies leading to nephrotic syndrome. With the increasing global population of older adults, it is important to act more proactively and make timely diagnosis through clinical and biopsy-guided data in this age group to streamline their treatment plan, since a higher rate of complications like acute renal failure, multi-morbidity, and relatively slower response to steroid and cyclophosphamide can present unique challenges in its management [4]. However, due to relatively better overall prognosis with stable remission and less frequent relapse rates, clinicians should intensify their efforts in making timely etiological diagnosis of nephrotic syndrome in older patients [5].

## Case presentation

Clinical case 1

A gentleman, 66 years old, with a known case of type 2 diabetes mellitus (on Metformin 500 mg twice daily), presented with a six-month history of painless, progressive, bilaterally symmetrical lower limb swelling. There was no history of any chest pain, shortness of breath, palpitations, abdominal pain or distension, hematemesis or melena, jaundice, frothuria, haematuria, change in urine output. There were no skin changes over the lower limbs. There were no recent use of non-steroidal anti-inflammatory drugs (NSAIDs) or any nephrotoxic medications. He had no significant weight loss, loss of appetite, night sweats, altered bowel habits, or hemoptysis. Physical examination showed stable hemodynamic parameters and bilateral pitting type of pedal oedema. Systemic examination was unremarkable. Laboratory workup has been shown in Table 1, which was significant for hypoalbuminemia and nephrotic range proteinuria.

**Table 1:** Laboratory investigations

Investigation	Patient Level	Reference Range
Haemoglobin (g/dL)	12.0	12-15
Total leukocyte count (× 10^3^/µL)	6.55	4-11
Platelet count (× 10^3^/µL)	270	150-400
Serum urea (mg/dL)	36.0	17-43
Serum creatinine (mg/dL)	0.90	0.55-1.02
Serum sodium (mmol/L)	137	136-146
Serum potassium (mmol/L)	3.9	3.5-5.1
Total bilirubin (mg/dL)	0.89	0.3-1.2
Direct bilirubin (mg/dL)	0.1	0-0.2
Aspartate transaminase (U/L)	36	0-35
Alanine transaminase (U/L)	30	0-35
Alkaline phosphatase (U/L)	124	30-120
Total Protein (g/dL)	4.6	6.6-8.3
Serum albumin (g/dL)	1.9	3.5-5.2
Serum globulin (g/dL)	2.7	2.5-3.2
Urine routine microscopy		
Pus cells (per high-power field)	Nil	Nil
Red blood cells (per high-power field)	Nil	Nil
Epithelial cells (per high-power field)	Nil	Nil
Protein (mg/dL)	3+ (approximately 1000)	Negative
Glucose (mg/dL)	Negative	Negative
24-hour urine protein/creatinine (mg/mg)	4000	<0.2

**Figure 1:**
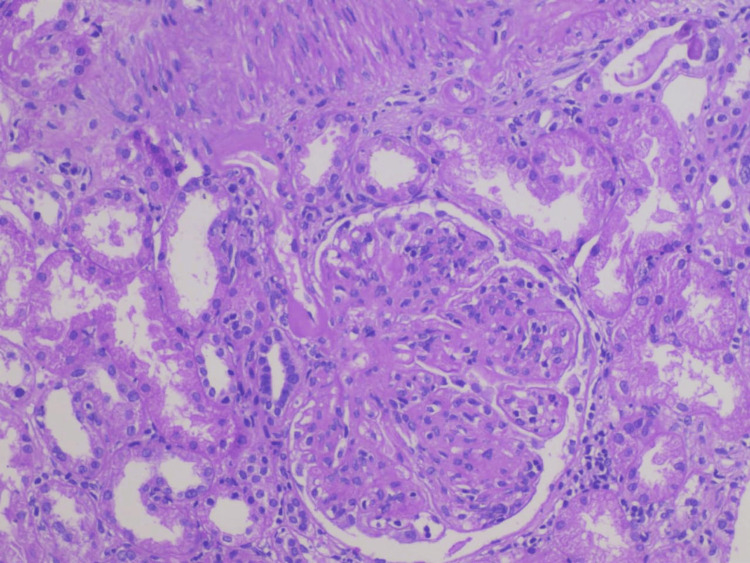
Pictomicrograph showing increased mesangial matrix expansion with sclerosis, interstitial fibrosis with chronic inflammation (H&E stain)

He underwent a renal biopsy, which showed glomerular tuft sclerosis with mesangial matrix expansion with IgA deposition on direct immunofluorescence (Figure 1).

A diagnosis of IgA nephropathy was made. He was started on a pulse dose of Methylprednisolone (1 g) intravenously for three days, followed by oral Prednisolone (0.5 mg/kgbody weight) on an alternate-day regimen. He was also started on oral Telmisartan (20 mg once daily) as an antiproteinuric measure. After 3 months of follow-up, in view of persistent proteinuria (3.5 g/mg of creatinine), he was started on Cyclophosphamide (2 g/kg body weight). Subsequent follow-up at 3 months showed an improvement in proteinuria (500 mg/mg of creatinine) and a reduction in lower limb swelling.

Clinical case 2

**Figure 2:**
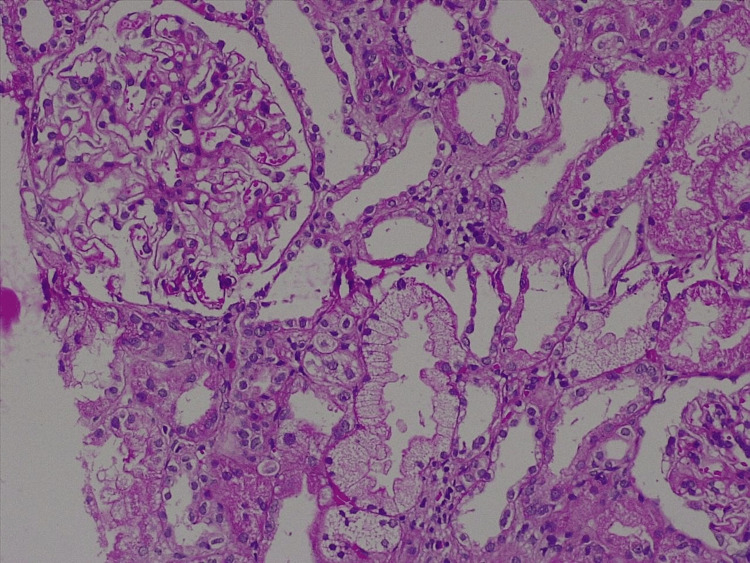
Pictomicrograph showing increased mesangial hypercellularity and PAS-positive mesangial matrix expansion and tubular atrophy PAS: Periodic Acid-Schiff

A gentleman, 78 years old, chronic smoker (smoking index 800), known case of chronic obstructive airway disease, presented with a history of anasarca for 8 months, which started with an insidious onset, painless, progressive, bilaterally symmetrical lower limb swelling, progressing eventually to involve the face and eventually to painless generalized abdominal distension over the past month. This was accompanied by exertional dyspnoea for one week before presentation. There was no history of any chest pain, palpitations, paroxysmal nocturnal dyspnoea, jaundice, melena or hematemesis, oliguria, frothuria. On physical examination, he was hemodynamically stable, with a puffy face and bilateral pitting pedal oedema. Systemic examination showed ascites with shifting dullness. His laboratory parameters (Table 2) showed hypoalbuminemia, nephrotic range proteinuria, hypertriglyceridemia. Diagnostic abdominal paracentesis was done, which showed a low serum-ascitic albumin gradient (SAAG=0.8). A provisional impression of nephrotic syndrome was made, and the etiological workup came positive for anti-phospholipase A2 receptor (Anti PLA2R) antibodies (80 RU/mL). Renal biopsy was performed subsequently, which showed features of membranous nephropathy (Figure 2) with diffuse granular positivity along glomerular capillary walls for anti PLA2R. A diagnosis of primary membranous nephropathy was made. Since he was in the low-risk category, he was initially started on oral Telmisartan 20 mg once daily with a low-dose oral diuretic (Torsemide 10 mg daily) with a high-protein diet. He was followed up at 1st and 3rd month with 24-hour urinary protein level and Anti PLA2R titres, which were stable (3.8 g/day and 85 RU/mL, respectively, at 3 months). However, at 6 months, his proteinuria slightly increased (4.2 g/g of creatinine), and his anti-PLA2R titres increased to 145 RU/mL. After consultation with the nephrology team, he was then started on intravenous Rituximab (1 g) therapy with subsequent repeat of the dose after 14 days. On further follow-up, his workup showed an improving proteinuria and a falling trend of PLA2R levels (20 RU/mL).

**Table 2:** Laboratory investigations HDL: high-density lipoprotein; LDL: low-density lipoprotein

Investigation	Patient level	Reference range
Haemoglobin (g/dL)	11.0	12-15
Total leukocyte count (×10^3^/µL)	7.00	4-11
Platelet count (×10^3^/µL)	350	150-400
Serum urea (mg/dL)	24.0	17-43
Serum creatinine (mg/dL)	0.78	0.55-1.02
Serum sodium (mmol/L)	140	136-146
Serum potassium (mmol/L)	4.2	3.5-5.1
Total bilirubin (mg/dL)	1.02	0.3-1.2
Direct bilirubin (mg/dL)	0.04	0-0.2
Aspartate transaminase (U/L)	28	0-35
Alanine transaminase (U/L)	24	0-35
Alkaline phosphatase (U/L)	109	30-120
Total protein (g/dL)	4.0	6.6-8.3
Serum albumin (g/dL)	2.2	3.5-5.2
Serum globulin (g/dL)	1.8	2.5-3.2
Urine routine microscopy		
Pus cells (per high-power field)	Nil	Nil
Red blood cells (per high-power field)	Nil	Nil
Epithelial cells (per high-power field)	Nil	Nil
Protein (mg/dL)	2+ (approximately 500)	Negative
Glucose (mg/dL)	Negative	Negative
24-hour urine protein/creatinine (mg/mg)	3500	<0.2
Total cholesterol (mg/dL)	198	123-200
Serum triglyceride (mg/dL)	380	50-150
HDL cholesterol (mg/dL)	38	40-60
LDL cholesterol (mg/dL)	112	100-130

Clinical case 3

**Figure 3:**
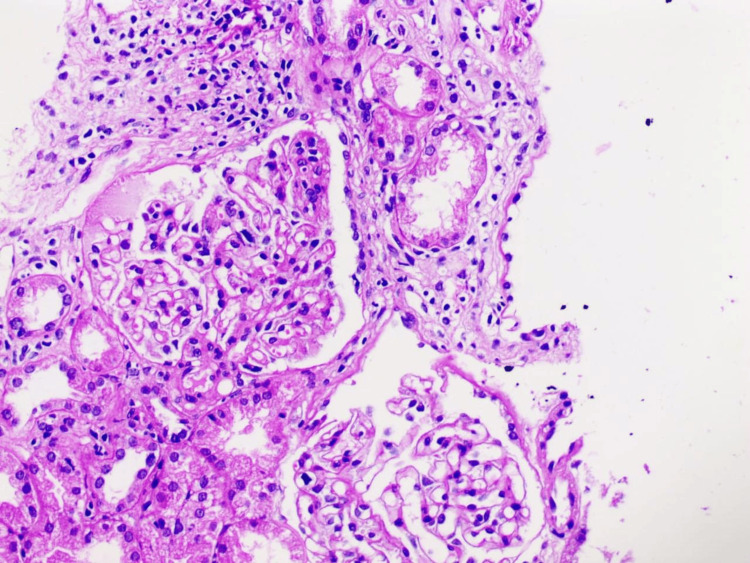
Pictomicrograph showing increased mesangial hypercellularity with Periodic Acid-Schiff (PAS) positive deposition, tubular atrophy and chronic inflammation

A lady, 75 years old, with a known case of systemic hypertension (on Amlodipine 10 mg daily), presented with anasarca for 5 months, which started with insidious onset, facial puffiness that progressed to painless, bilaterally symmetrical lower limb swelling and finally generalised abdominal distension for one month. This was associated with frothuria and progressively reduced urinary output for fifteen days. There was no associated history of any palpitations, chest pain, paroxysmal nocturnal dyspnoea, jaundice, melena or hematemesis. There was no preceding history of alternative medication or NSAIDs intake, fever, dysuria or haematuria. Physical examination showed stable hemodynamic parameters, pitting pedal oedema and in systemic examination, ascites with shifting dullness was present (Table 3) showed normocytic normochromic anaemia, hypoalbuminemia, and azotaemia with normal electrolyte levels. The total 24-hour urinary protein quantification showed nephrotic range proteinuria. Ascitic fluid workup was suggestive of low SAAG ascites (0.9). Her anti-PLA2R antibody was negative. A renal biopsy was performed, which showed segmental glomerular tuft sclerosis with intraglomerular hyalinosis consistent with focal and segmental glomerular sclerosis (FSGS) without any significant staining on direct immunofluorescence (Figure 3).

**Table 3:** Laboratory investigations

Investigation	Patient level	Reference range
Haemoglobin (g/dL)	10.0	12-15
Total leukocyte count (×10^3^/µL)	8.32	4-11
Platelet count (×10^3^/µL)	186	150-400
Mean corpuscular haemoglobin (pg)	29.2	27-32
Mean corpuscular volume (fL)	87.1	78-98
Serum urea (mg/dL)	48.0	17-43
Serum creatinine (mg/dL)	2.00	0.55-1.02
Serum sodium (mmol/L)	142	136-146
Serum potassium (mmol/L)	4.5	3.5-5.1
Total bilirubin (mg/dL)	0.96	0.3-1.2
Direct bilirubin (mg/dL)	0.23	0-0.2
Aspartate transaminase (U/L)	24	0-35
Alanine transaminase (U/L)	32	0-35
Alkaline phosphatase (U/L)	48	30-120
Total protein (g/dL)	3.6	6.6-8.3
Serum albumin (g/dL)	1.8	3.5-5.2
Serum globulin (g/dL)	1.8	2.5-3.2
Urine routine microscopy		
Pus cells (per high-power field)	Nil	Nil
Red blood cells (per high-power field)	Nil	Nil
Epithelial cells (per high-power field)	Nil	Nil
Protein (mg/dL)	3+ (approximately 1000)	Negative
Glucose (mg/dL)	Negative	Negative
24-hour urine protein/creatinine (mg/mg)	4000	<0.2
Total cholesterol (mg/dL)	91	123-200
Serum triglyceride (mg/dL)	117	50-150
HDL cholesterol (mg/dL)	10	40-60
LDL cholesterol (mg/dL)	63	100-130

Her renal dysfunction initially was managed conservatively with careful fluid management and oral bicarbonate replacement in view of metabolic acidosis. Intravenous human albumin was given along with a high-protein diet in view of severe hypoalbuminemia. Once her renal function stabilized, she was started on a low-dose oral diuretic (Torsemide 5 mg daily) in view of anasarca with careful titration based on response. After the biopsy report, with nephrology consultation, she was started on oral Prednisolone 60 g daily with a tapering regimen over 2 months. At 1-month follow-up, her albumin levels had increased slightly (2.4 g/dL) with stable renal function and resolving anasarca.

## Discussion

The spectrum of adult-onset nephrotic syndrome consists of a more diverse group of aetiologies, including primary and secondary conditions. With increasing longevity, the proportion of older adults with nephrotic syndrome is gradually increasing. Glomerulopathies contribute to almost 25% cases of renal failure in older adults [6]. Although they do not differ from the general population from a clinical standpoint, there is a difference in underlying aetiologies. Renal biopsy continues to be an essential investigation to diagnose these patients and plan their treatment. Although limited, the existing data says that performing prompt renal biopsy in these older patients can help plan appropriate immunosuppressant therapy with better survival [7]. 

According to the available literature, the commonest cause of primary nephrotic syndrome in older adults is Membranous nephropathy (MN), contributing to as high as 35% cases of nephrotic syndrome in them [1, 8-9]. Studies have shown that MN in older patients show up to 55.2% complete remission and 62.1% partial remission on prednisolone and low-dose cyclophosphamide [9]. However, there has been an increased incidence of renal dysfunction (17.2%) and histopathological deterioration even after complete remission reported in studies in MN patients who are on long-term anti-proteinuric treatments or cyclophosphamide [9]. With an increase in the older population, MN is slowly becoming one of the major causes of chronic kidney disease (CKD) in older adults [10]. However, almost 75% cases of MN in older adults are primary (like our case 2) [11], it is important to rule out any secondary causes like hepatitis B,C, Drugs like NSAIDs, etc., since treating those often can lead to resolution of symptoms.

Another important entity of nephrotic syndrome in older adults is FSGS, with studies reporting its prevalence from 9-12% [1,12]. In contrast to MN, which has an excellent prognosis, FSGS is likely to evolve into CKD and the need for renal replacement therapy [12]. FSGS has also been seen to be associated with extraglomerular involvement, with studies reporting 70-80% of patients with FSGS having tubulo-interstitial and vascular involvement (like in our case 3) [12]. This is important since extraglomerular involvement in these glomerulopathies has been associated with shorter renal survival and poorer prognosis [13]. Studies have shown conflicting evidence regarding response to immunosuppressants and complete remission in patients with FSGS [8,14]. This can be attributed to the underlying pathogenesis of these FSGS lesions, where the Kidney Disease Improving Global Outcome (KDIGO) has recommended that steroids and immunosuppressants should only be considered in idiopathic FSGS with clinical features of nephrotic syndrome [15]. Excluding the long list of secondary causes may be difficult and confusing, especially in multimorbid older adults, where completely ruling out the possibility of superimposed idiopathic FSGS might not be possible. However, it is important to decide which patients should receive steroids and immunosuppressants since remission dramatically improves their overall prognosis [16].

However, the varied spectrum of nephrotic syndrome in older adults also comprises Minimal Change Disease (MCD), Amyloidosis, Membranoproliferative glomerulonephritis (MPGN) and rarely IgA nephropathy. A study reported that out of 44 elderly nephrotic syndrome patients, 11.4% had MCD, 22.7% had renal amyloidosis, and 2.3% had IgA nephropathy and MPGN each [12]. IgA nephropathy is an emerging glomerulopathy among older adults with a prevalence of 4-10% across the world [17,18]. Although there is still much to be researched regarding its course in older patients, existing literature suggests that IgA nephropathy is associated with a higher grade of proteinuria, a greater degree of tubulo-interstitial sclerosis and faster progression of renal impairment despite treatment [19].

Like the three cases described here, it is often difficult to differentiate between these aetiologies only based on clinical history or laboratory findings without a renal biopsy. There has been a general reluctance for biopsy in older patients on grounds of advanced age, fear of procedure-related complications and following a more conservative treatment approach in them. Additionally, age-related changes in pharmacokinetic and pharmacodynamic properties may lead to increased treatment-related side effects [19]. Despite these, studies have shown that elderly nephrotic syndrome patients who have undergone biopsy are associated with improved treatment outcomes, like a higher rate of complete remission, better survival treatment without any significant increase in complications or side effects [6,14].

## Conclusions

Older adults with nephrotic syndrome generally have an overall good prognosis. Renal biopsy is a generally safe procedure in older adults without any increased risk of complications and should not be withheld only on the grounds of age alone. Prompt renal biopsy can help in starting targeted therapy for an excellent outcome in these patients and predicting renal and patient survival. Treatment for older adults should be individualized taking into consideration the multiple age-related factors and comorbidities for better holistic care.
